# Rheumatoid Arthritis Related Interstitial Lung Disease: Patterns of High-resolution Computed Tomography

**DOI:** 10.7759/cureus.6875

**Published:** 2020-02-04

**Authors:** Mahesh Gautam, Mah Jabeen Masood, Sadaf Arooj, Mufazzal-e-Haque Mahmud, Muhammad Umer Mukhtar

**Affiliations:** 1 Radiology, Nobel Medical College, Biratnagar, NPL; 2 Radiology, King Edward Medical University, Lahore, PAK; 3 Rheumatology and Immunology, Shaikh Zayed Hospital, Lahore, PAK; 4 Medicine, King Edward Medical University, Lahore, PAK

**Keywords:** rheumatoid arthritis, interstitial lung disease, hrct

## Abstract

Background and aim

Rheumatoid arthritis (RA) is a chronic inflammatory systemic disease characterized by bilateral involvement of mostly small joints of hands and feet. There can be various extra-articular manifestations of the disease including lung parenchymal disease. Pulmonary involvement in RA patients leads to increased morbidity and mortality. The overall burden of RA related pulmonary disease is underestimated due to the limitation of resources in underdeveloped countries. High-resolution computed tomography (HRCT) is an important tool used to diagnose different abnormalities in RA related interstitial lung disease (ILD). The objective of the study was to evaluate HRCT findings in patients of RA related ILD and categorize the radiological findings according to clinical findings.

Method

This descriptive prospective observational study was conducted at Mayo Hospital, Lahore from June 2014 to June 2015. Patients of RA suspected of lung disease after selection underwent HRCT chest on 128-slice Hitachi CT scanner (Hitachi Global, Tokyo, Japan) in the radiology department. Images were reconstructed and evaluated by experienced radiologists. Findings were recorded on a questionnaire. Data was analyzed on SPSS version 21 (IBM Corp, Armonk, US).

Results

Out of the 54 patients scanned, interlobular septal thickening was the most common finding found in 22 of the patients. Ground-glass opacification was recognized in 21 patients, honeycombing in nine and bronchiectasis in two patients. Regarding zonal predilection of disease pattern, lower zones of lungs were found involved in most of the cases. The disease was found to be bilateral in 15 patients. Based on these findings, usual interstitial pneumonitis (UIP) was diagnosed in six patients and non-specific interstitial pneumonitis (NSIP) in 14 others.

Conclusion

This study concluded that HRCT images are very useful in diagnosing interstitial lung disease related to rheumatoid arthritis.

## Introduction

Rheumatoid arthritis (RA) is a chronic inflammatory systemic disease exhibiting clinical signs and symptoms of predominantly joint disease [1-3]. This disease is characterized by symmetrical bilateral involvement of mostly small joints of hand and feet; however as it leads to chronic synovitis, all joints may be involved [4-5]. There can be various extra-articular manifestations (EAM) of the disease e.g. upper airway, lower airway, pleural, vascular and lung parenchymal disease, etc. [6-8]. The overall burden of RA related pulmonary disease is underestimated due to the limitation of resources in underdeveloped countries [9]. Prevalence of interstitial lung disease (ILD) was found to be 97.9% per 100000 with more of the secondary ILDs than primary ILD. is around 19-44% [10]. Pulmonary involvement seen in RA patients has high clinical significance as it leads to increased morbidity and mortality [11]. The most important characteristic of RA related pulmonary disease is that almost all anatomical parts of the lung are prone to RA related tissue injury [12]. The overall risk of having ILD in RA patients is 19.2% as compared to the risk of having ILD in the common population [13]. ILD is a spectrum of pulmonary diseases that involve all parts of pulmonary bronchovascular units, including alveolar epithelium, capillary endothelium, alveoli, perivascular connective tissue, and perilymphatic tissues. RA is only second to systemic sclerosis as far as the incidence of ILD in connective tissue disorders is concerned [14]. The natural history of ILD is not very well defined in patients. Complications may arise themself or secondary to immunosuppressive drug treatment. High-resolution computed tomography (HRCT), that emerged during the past decade, is an important tool to diagnose the different abnormalities in RA related ILD.

## Materials and methods

From June 2014 to June 2015, 54 patients were selected from the outpatient department of Mayo Hospital, Lahore, with RA related pulmonary disease, according to American College of Radiology (ACR) criteria 2010 [15]. The complete history was taken, and patients with co-morbidities like pulmonary tuberculosis, chronic obstructive pulmonary disease (COPD), and lung masses were excluded after evaluating chest X-rays. HRCT chest was performed using a multislice multidetector scanner. Axial images were acquired in a supine position, taking complete deep inspiration. Images were taken using a 0.5 m slice thickness with at least 1 cm slice interval. Image reconstruction was done using a bone algorithm.

## Results

Altogether 54 cases were studied to complete the sample size of the project. Out of the 54 patients, 18 (33.33%) were male, while 36 (66.67%) were female. The mean age of the patient was 44.17± 11.315 years, with the minimum age being 15 years and the maximum age being 65 years. Patients had a variable presentation, and the commonest presentation to the hospital was exertional dyspnea (Table 1).

**Table 1:** Frequency distribution of the respiratory symptoms

Symptoms	Present	Duration in months
Cough	7 (13%)	3-6 months
Exertional dyspnea	20 (37%)	1-3 months
Wheezing	0	0
Phlegm	3 (5.6%)	1 month

Regarding the findings of HRCT, interlobular septal thickening was the most common finding, present in 22 (40.7%) patients. Similarly, ground-glass opacity (GGO) was present in 21 (38.9%) patients, while nine (16.7%) patients had honeycombing (Table 2).

**Table 2:** Descriptive statistics of the patients with early rheumatoid arthritis using HRCT HRCT - high-resolution computed tomography

HRCT Findings	Frequency %
Ground-glass attenuation	21 (38.9%)
Honeycombing	9 (16.7%)
Interlobular septal thickening	21 (38.9%)
Nodule	0
Air trapping	1 (1.9%)
Bronchiectasis	1 (1.9%)
Traction bronchiectasis	15 (27.8%)
Reticular shadowing	22 (40.7%)
Mosaic perfusion	2 (3.7%)
Architectural distortion	3 (5.6%)

Bronchiectasis was present in two (3.7%) patients, whereas traction bronchiectasis was present in 17 (31.5%). Air trapping was present in one (1.9%), mosaic perfusion in two (3.7%), and architectural distortion in three (5.6%) patients. GGO, interlobular septal thickening and reticular shadowing, honeycombing, air trapping, mosaic perfusion, and air trapping were found to involve both the lungs. Fifteen (27.8%) patients had bilateral symmetrical traction bronchiectasis, whereas one (1.9%) patient had traction bronchiectasis involving right lung and one (1.9%) patient had traction bronchiectasis involving left lung only (Table 3). Similarly, one (1.9%) had unilateral bronchiectasis involving the right lung, and one (1.9%) patient had bronchiectasis involving the left lung.

**Table 3:** Descriptive statistics of the HRCT findings in unilateral and bilateral involvement of the right and left lung HRCT- high-resolution computed tomography

Findings	Unilateral right lung involvement	Unilateral left lung involvement	Bilateral involvement
Ground-glass attenuation	0	0	21 (38.9%)
Honeycombing	0	0	9 (16.7%)
Interlobular septal thickening	0	0	22 (40.7%)
Nodule	0	0	0
Air trapping	0	0	1 (1.9%)
Bronchiectasis	1 (1.9%)	1 (1.9%)	0
Traction bronchiectasis	1 (1.9%)	1 (1.9%)	15 (27.8%)
Reticular shadowing	0	0	22 (40.7%)
Air space opacity	0	0	0
Emphysema	0	0	0
Cysts	0	0	0
Tree-in-bud appearance	0	0	0
Crazy-paving appearance	0	0	0
Mosaic perfusion	0	2 (3.7%)	0
Architectural distortion	0	3 (5.6%)	0
Thickening of bronchovascular bundle	0	0	0

Regarding the zone involvement, the lower zone was found to be more frequently involved (Table 4). GGO was found to involve lower zone in 14 (25.9%) patients, one (1.9%) patient had involvement of mid zone, five (9.3%) patients had involvement of the middle and lower zone and one (1.9%) patient had involvement of all three zones, i.e., upper, mid and lower. Honeycombing was present in the lower zone in six (11.1%) patients, two (3.7%) patients had honeycombing in the middle and lower zone, and one (1.9%) had honeycombing involving all three zones. Thirteen (24.1%) patients had interlobular septal thickening involving mid and lower zone, eight (14.8%) had interlobular septal thickening in the lower zone, and one (1.9%) had interlobular septal thickening involving all three zones.

**Table 4:** Descriptive statistics of the HRCT findings in term of zone involved among patients with early rheumatoid arthritis HRCT - high-resolution computed tomography

HRCT finding	Right lung					Left lung				
	upper	mid	lower	mid and lower	upper mid and lower	upper	mid	lower	mid and lower	upper mid and lower
Ground-glass Opacity	0	1 (4.8%)	14 (66.7%)	5 (23.8%)	1 (4.8%)	1 (4.8%)	14 (66.7%)	5 (23.8%)	1 (4.8%)	
Honeycombing	0	0	6 (66.7%)	2 (22.2%)	1 (11.1%)	0	0	6 (66.7%)	2 (22.2%)	1 (11.1%)
Interlobular septal thickening	0	0	8 (36.0%)	13 (59.0%)	1 (4.0%)	0	0	8 (36.0%)	13 (59.0%)	1 (4.0%)
Nodule	0	0	0	0	0	0	0	0	0	0
Air trapping	0	0	1 (100%)	0	0	0	0	1 (100%)	0	0
Bronchiectasis	0	0	0	0	1 (100%)	0	0	1 (100%)	0	0
Traction bronchiectasis	0	1 (6.7%)	1 (100%)	0	0	0	1 (6.7%)	1 (100%)	0	0
Reticular shadowing	0	0	16 (68.2%)	6 (27.3%)	1 (4.5%)	0	0	16 (68.2%)	6 (27.3%)	1 (4.5%)

Based on these patterns of involvement of the lung, usual interstitial pneumonia (UIP) was diagnosed in six (11.1%) patients (Table 5). Similarly, non-specific interstitial pneumonia (NSIP) was diagnosed in 14 (25.9%) of the patients. Findings of HRCT that did not fall under any defined category were labeled as others, as were present in five (9.3%) patients.

**Table 5:** Frequency distribution of the HRCT diagnosis in patients with early rheumatoid arthritis NSIP - non-specific interstitial pneumonia

Diagnosis	Frequency	Percent	Valid percent	Cumulative percent
Normal	29	53.7	53.7	53.7
Usual interstitial pneumonia	6	11.1	11.1	64.8
NSIP	14	25.9	25.9	90.7
Others	5	9.3	9.3	100.0
Total	54	100.0	100.0	

## Discussion

The present study indicates that females are at increased risk of RA with the statistical odds of (66.67% vs. 33.3%). The results of this study are comparable with the study by Gabbay E et al. that demonstrated females to be at an increased risk of RA (69.44%) [16]. El Khalik KA et al. found that RA was more common in females than in males (79.4% vs. 20.58%) [17]. This disparity may be due to bias in the selection of women. The present study provides evidence that RA patients were older individuals of 44.17 ±11.315 years. Gabbay E et al. demonstrated that RA was more commonly found in older patients 51.8 ±16.0 years [16]. Affara NK et al. examined RA patients to be 52.6 ± 5.1 years old [18]. The discrepancy in the mean age of the patients in our study may be present due to the small sample size.

**Figure 1:**
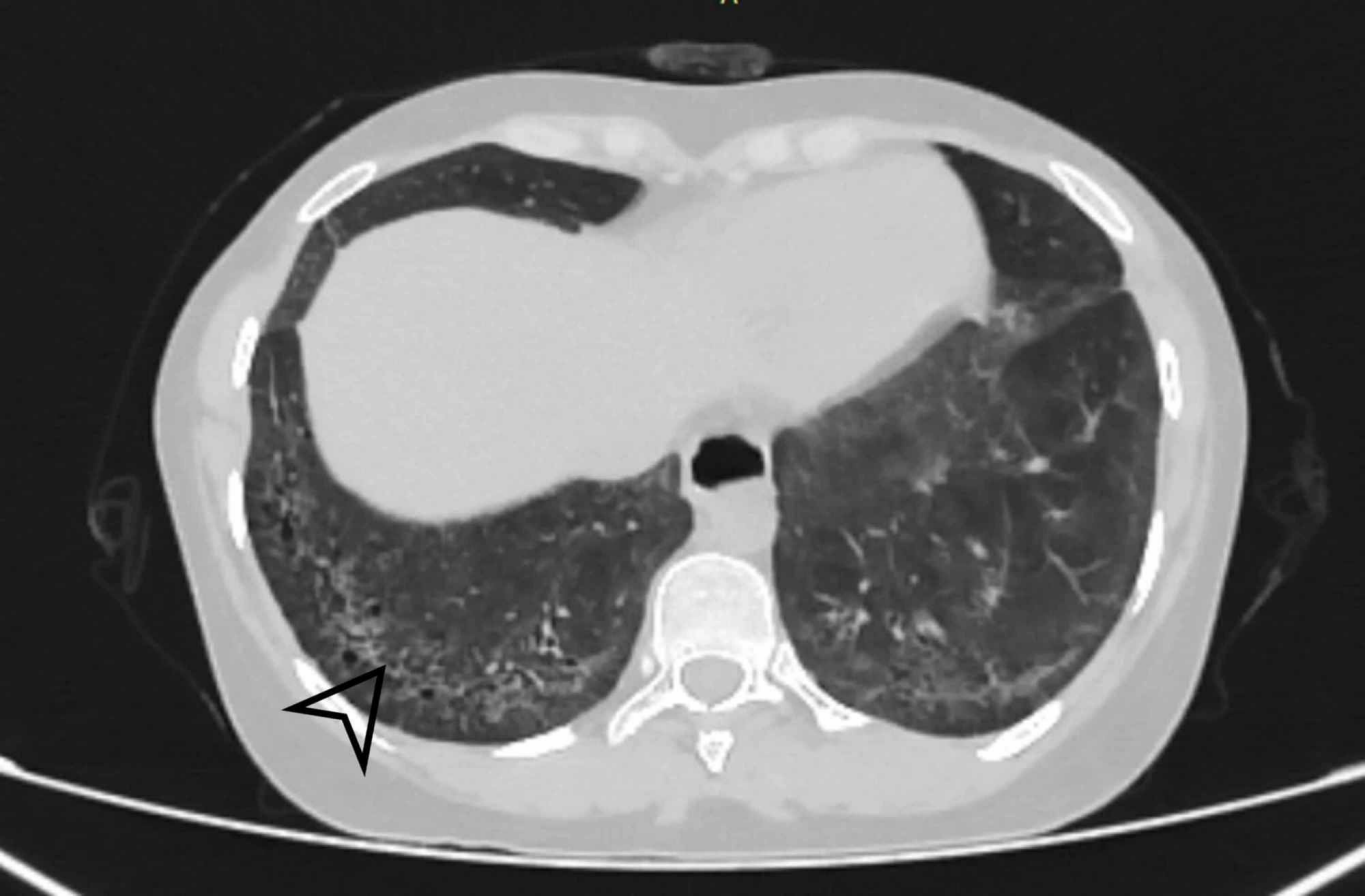
Axial section revealing bilateral basal intralobular septal thickening with sparing of subpleural areas Black arrowhead shows Interlobular septal thickening.

**Figure 2:**
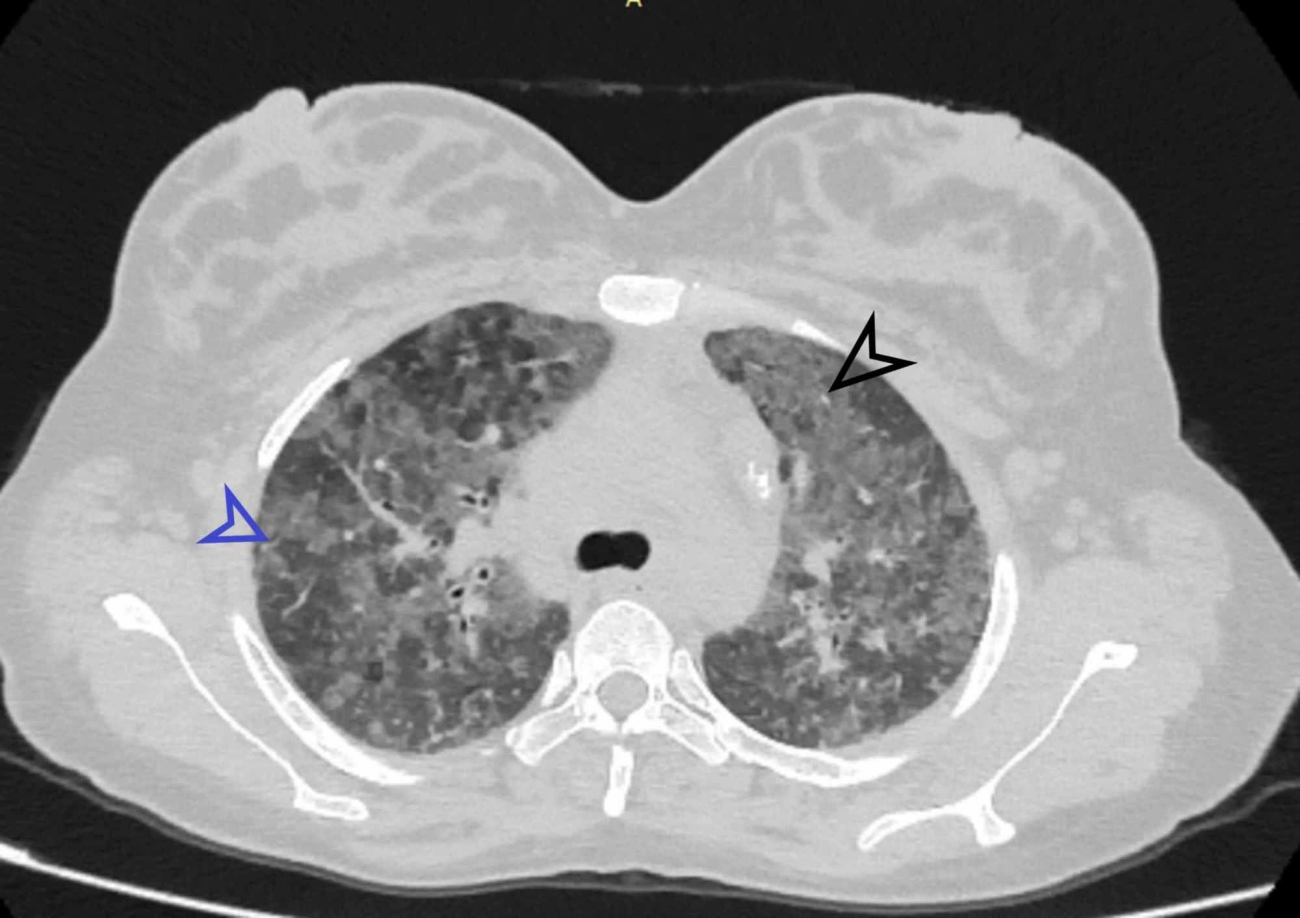
Patchy areas of GGO and interlobular septal thickening involving bilateral upper lobes Black arrowhead indicates the ground-glass opacity (GGO). Blue arrowhead shows interlobular septal thickening.

Affara NK et al. examined HRCT abnormalities in their study, i.e., GGO was present in 30%, air space opacification (AS) was present in 13.3%, mixed AS/GGO was present in 13.3%, honeycombing in 10%, septal thickening in 26.7% and traction bronchiectasis in 11.7% of the subjects [18]. In contrast with these studies, our study reported interlobular septal thickening as the most common HRCT finding, present in 22 (40.7%) patients and illustrated in Figures 1 and 2.

**Figure 3:**
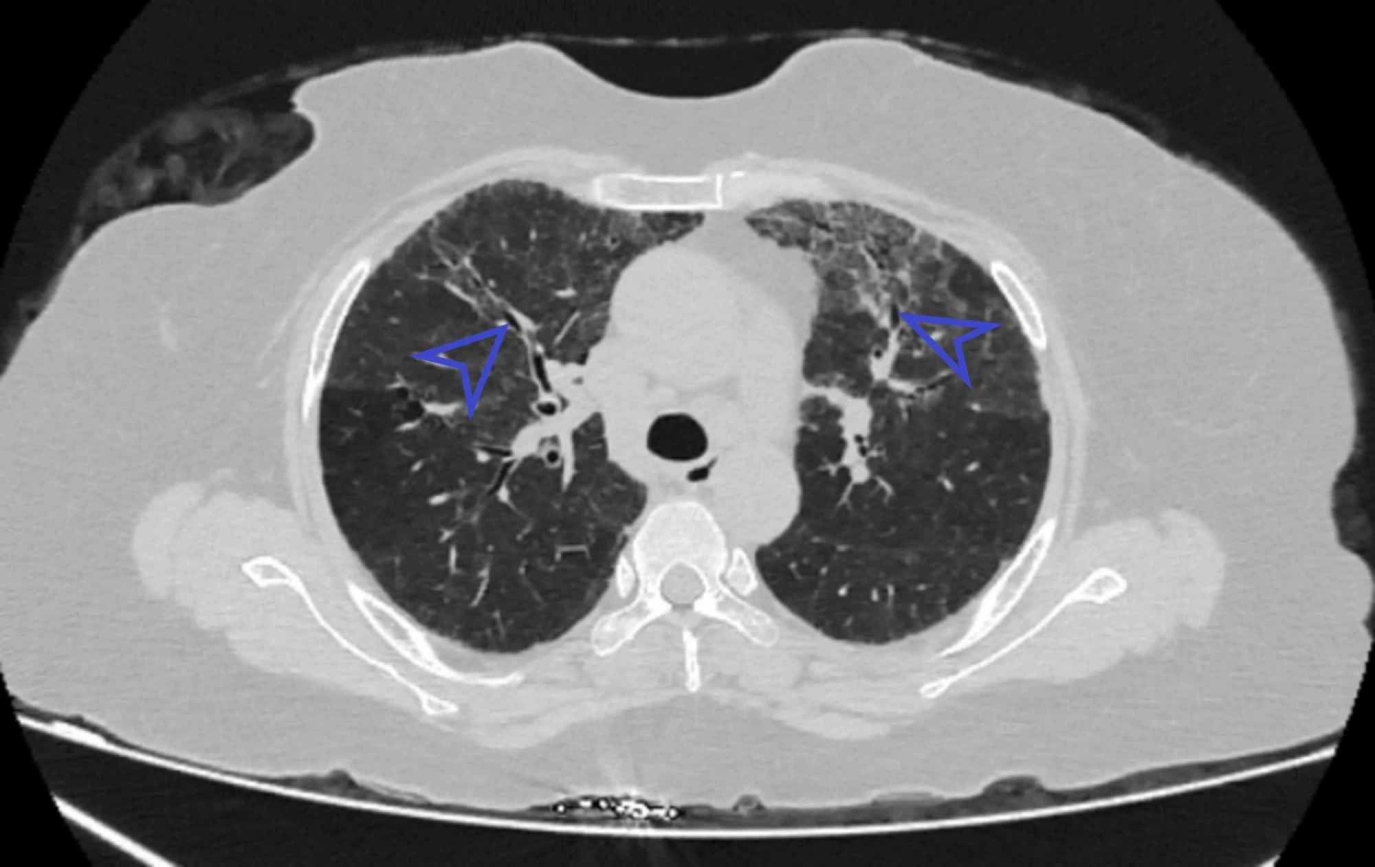
Axial section showing bronchiectasis Blue arrowhead indicates bronchiectasis.

Similarly, GGO was present in 21 (38.9%) patients (Figure 2), nine (16.7%) patients had honeycombing. Bronchiectasis was present in two (3.7%), whereas traction bronchiectasis was present in 17 (31.5%) such as shown in Figure 3. Air trapping was present in one (1.9%), two (3.7%) patients had mosaic perfusion, and three (5.6%) patients had architectural distortion.

Metafratzi ZM et al. .used normal healthy individuals as control against known patients of RA [19]. They used a semi-quantitative grading system, which has also been described in the past. The presence and extent of findings on HRCT chest were coded according to lung zones bilaterally, making a total of six zones. Only air trapping was given a score of eight on its presence on paired inspiratory and expiratory images. The control subjects showed minimal findings with scores of less than 3.6. The most common findings were air trapping and bronchiectasis. The abnormalities noticed in patients were equal to a score of 5.2 (moderate in severity) with only air trapping having a score of 14 (maximum severity). Other findings were bronchiectasis, bronchial wall thickening, macro nodules, and GGO.

**Figure 4:**
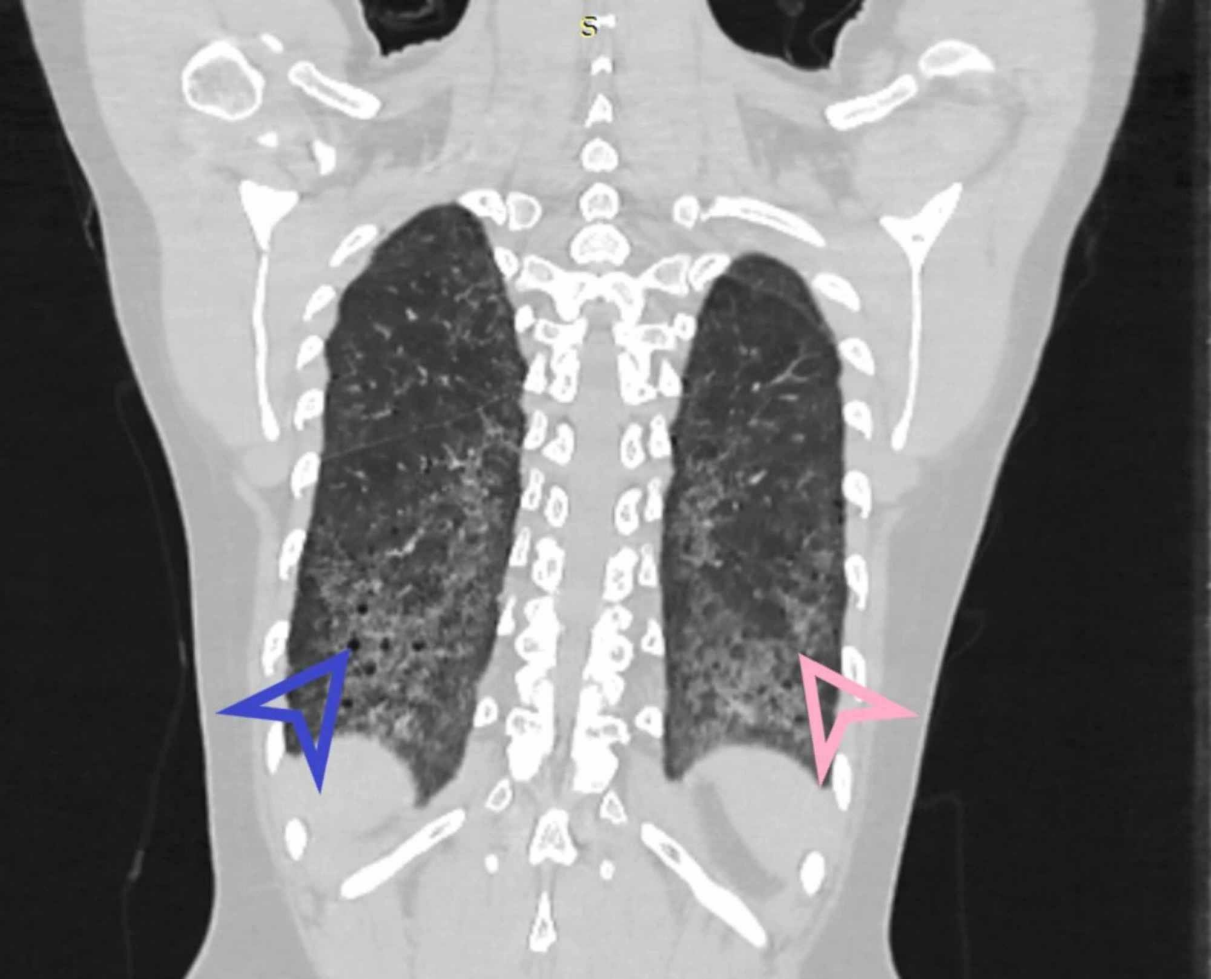
Coronal HRCT showing basal predominance of fibrosis and honeycombing in UIP HRCT - high-resolution computed tomography; UIP - usual interstitial pneumonia Blue arrowhead indicates honeycombing. Pink arrowhead shows fibrosis.

**Figure 5:**
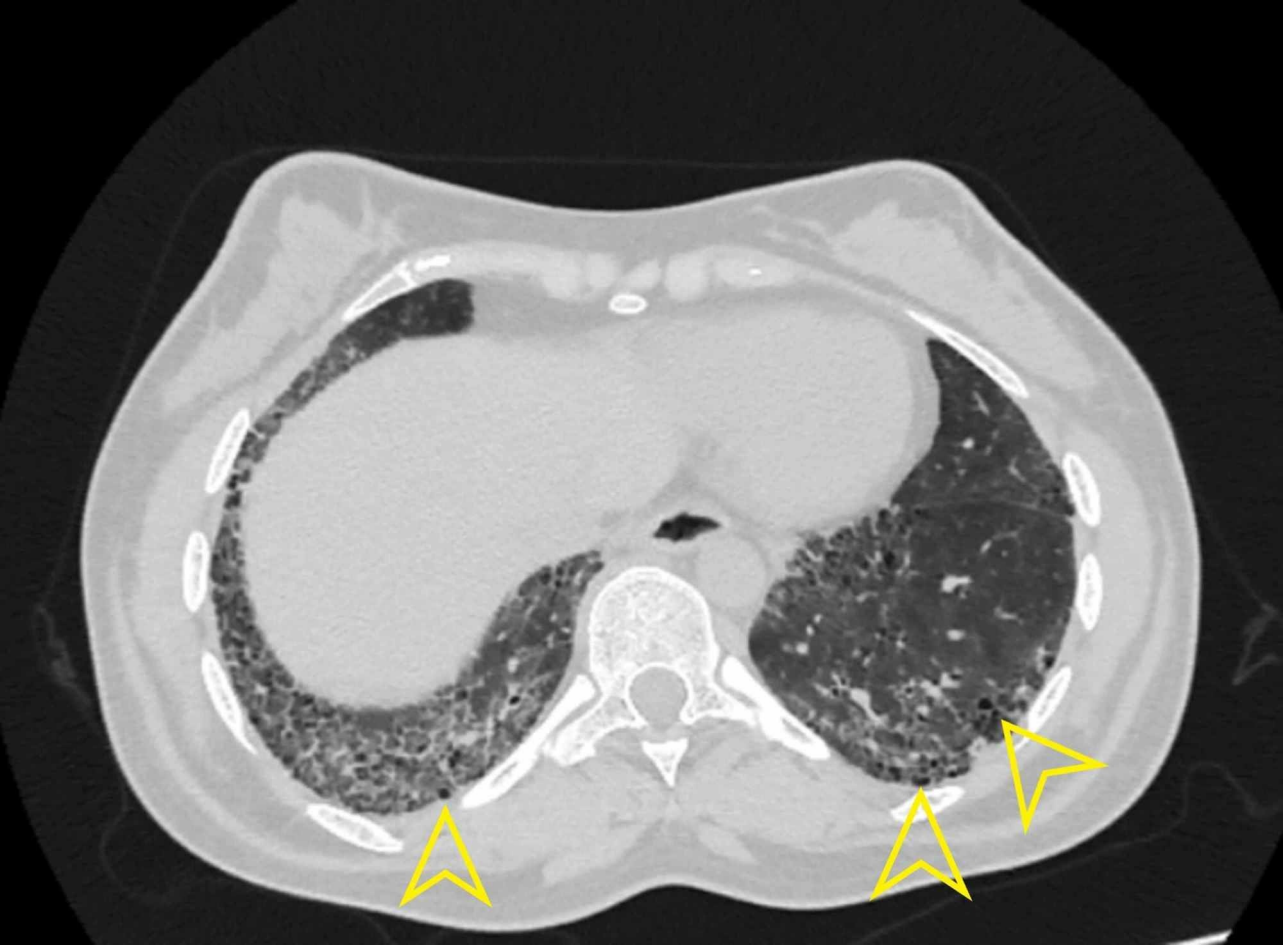
Axial section high-resolution computed tomography showing UIP Yellow arrowhead indicating honeycombing in usual interstitial pneumonia (UIP).

**Figure 6:**
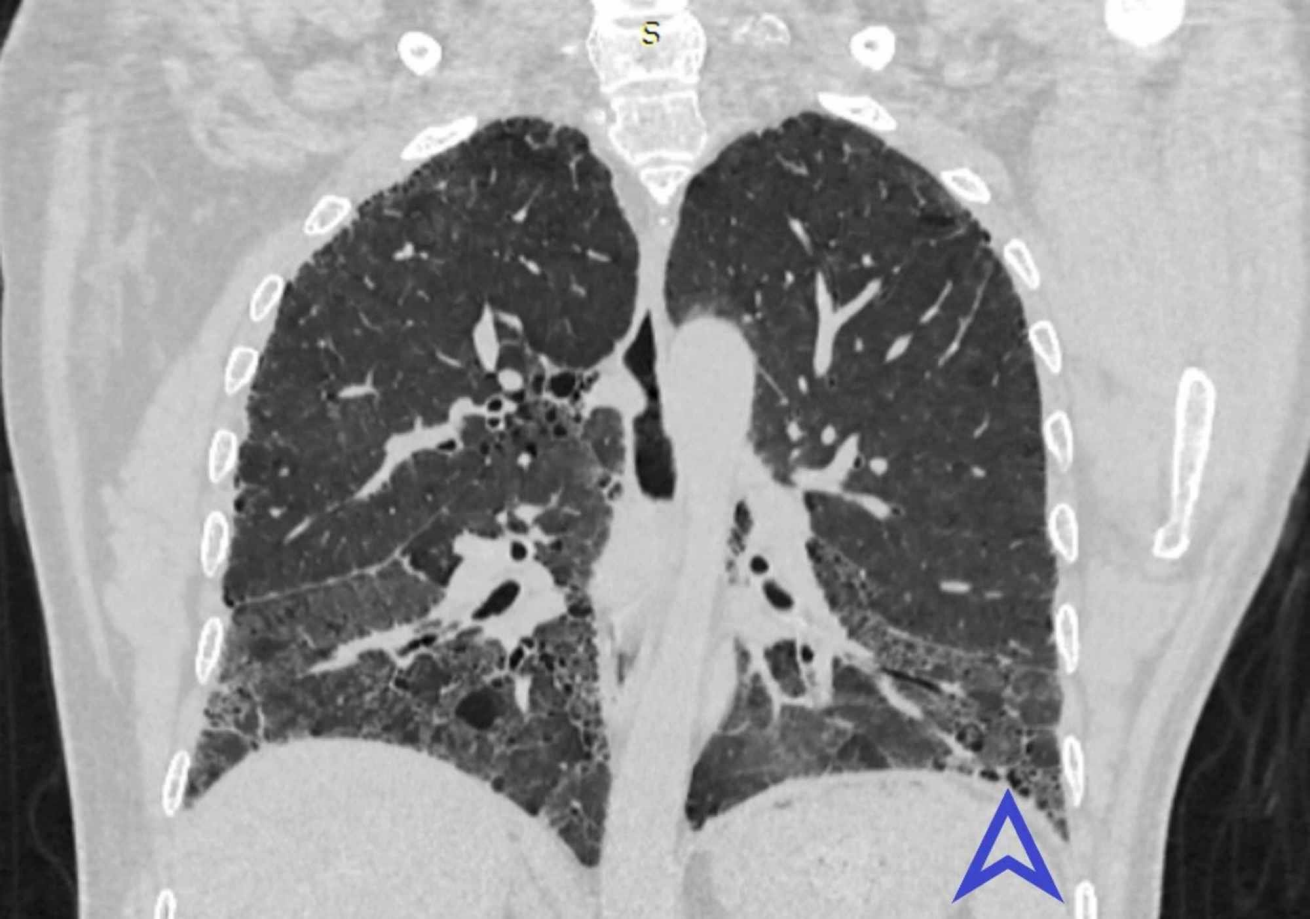
Coronal view HRCT shows bilateral symmetrical fibrosis in lower lobes HRCT - high-resolution computed tomography Blue arrowhead shows honeycombing.

Chansakul TN, in their study, concluded that traction bronchiectasis and the extent of honeycombing (as our earlier findings illustrated in Figures 4-6) were strongly associated with morbidity and mortality [20]. RA related ILD carried a bad prognosis when they compared HRCT findings to pulmonary function tests. They also completely evaluated patients’ intrathoracic noncardiac findings on CT scans in terms of pleural, parenchymal, vascular disease as well as drug-related complications and opportunistic infections. They found that UIP (illustrated in Figure 5) was commoner than NSIP. There was more overlap between NSIP and UIP patterns.

## Conclusions

Multiple pulmonary manifestations of RA are well known. Investigation for ILD is mandatory for evaluating the clinical progression of RA. HRCT detects subtle abnormalities in RA patients even without respiratory symptoms. Thus, HRCT is the investigation of choice to delineate in detail the bronchovascular involvement and disease patterns of the lungs in patients of RA.
